# The New Insight into the Role of Antimicrobial Proteins-Alarmins in the Immunopathogenesis of Psoriasis

**DOI:** 10.1155/2014/628289

**Published:** 2014-05-08

**Authors:** A. Batycka-Baran, J. Maj, R. Wolf, J. C. Szepietowski

**Affiliations:** ^1^Department of Dermatology, Venereology and Allergology, Wroclaw Medical University, ul. Chalubinskiego 1, 50-368 Wroclaw, Poland; ^2^Department of Dermatology and Allergology, Ludwig-Maximilian University, Thalkirchner Straße 48, 80539 Munich, Germany

## Abstract

The pathognesis of psoriasis still remains not fully elucidated. Recent advances favor the idea that interactions between innate and adaptive immune response drive inflammatory process in this disease. Innate antimicrobial peptides and proteins (AMPs) are diverse group of small molecules that provide the first line of defense against invading pathogens. In recent years, the novel functions of AMPs have been identified. There are three subclasses among AMPs that have gained the special interest as a potentially important player in the pathogenesis of psoriasis: cathelicidin, S100 proteins, and defensins. These AMPs have been shown to modulate and trigger host immune response in psoriasis acting as interplayer between innate and adaptive immune mechanisms. Overexpressed in psoriatic lesions, they prime immune cells for enhanced production of proinflammatory mediators and act as chemoattractant for leukocytes. Therefore, the novel term describing AMPs alarmins has been suggested. As multifunctional player in pathogenesis of psoriasis, AMPs may constitute potential target for therapeutic interventions. However, further investigations are required to establish the methods of downregulation of the aberrant proinflammatory functions of AMPs without increasing the risk of infections.

## 1. Introduction

Psoriasis is a chronic immune-mediated inflammatory skin disease that affects approximately 1–3% of the population worldwide and significantly impairs patients' quality of life. Psoriatic skin lesions are sharply demarcated scaly plaques. They are histologically characterized by epidermal changes, inflammatory skin infiltrate, and increased angiogenesis. The pathogenesis of psoriasis is multifactorial and remains not fully elucidated. It is thought to result from the combination of genetic, environmental, and immunological factors [1–5]. Psoriasis is currently regarded as T-cell mediated inflammatory skin disease with certain systemic consequences, including increased cardiovascular risk and diabetes. Considerable progress in the understanding of the psoriasis immunopathogenesis has been resulting in the development of targeted systemic immunotherapies [4, 5]. Despite the crucial role of T helper 1 (Th1), Th17, Th22 cells and associated cytokines in psoriasis, recent studies highlight the significant role of innate immune mechanisms [3–8]. Most current concepts favor the idea that cell- and mediator-dependent interactions between innate and adaptive immune system together with keratinocyte defect may drive inflammatory process in this disease. The keratinocytes within psoriatic plaques show abnormal proliferation and differentiation and likely influence T-cells and other immune cells by production of various proinflammatory mediators. Recent evidence also underlines the role of other innate immune cells, such as dendritic cells in psoriasis [3, 7–9]. The characteristic abnormality of psoriatic skin lesions is excessive production of innate antimicrobial peptides and proteins (AMPs) [10–15].

Antimicrobial peptides and proteins (AMPs) are diverse group of small molecules (12–100 amino acid residues) that constitute primary effector system of innate immunity. They provide the first line of defense against pathogens. Phylogenetically old, they may present similar sequences in various species. They lack the specificity of antigen recognition and any characteristic biologically active amino acid sequence but possess certain common structural features responsible for their antimicrobial activity. AMPs contain positive charge, relatively hydrophobic and amphipathic structure that allow them to interact with negatively charged phospholipids of microbial membrane. This results in pores formation and antimicrobial activity. AMPs, produced in response to danger, are able to kill in short time wide spectrum of the microbes, such as bacteria, fungi, viruses, or protozoa [15–20].

In 1990s the AMPs were discovered to be expressed in human skin [11, 12]. They may be produced by both resident skin cells and infiltrating immune cells, either constitutively or in response to danger, such as infection, trauma, wound healing, or chronic inflammation. Keratinocytes and phagocytes are the main source of AMPs in the skin [15, 20–24]. Although the integral role of these molecules is to kill pathogenic microorganism, in recent year the novel functions of AMPs, far beyond their antimicrobial activity, have been identified. In vertebrate AMPs seem to maintain their biological relevance acting through variety of mechanisms and constitute important part of skin immune system. They control host physiological functions, such as angiogenesis, wound healing, and inflammation. Several AMPs have been shown to modulate host immune-mediated inflammatory response acting as chemotactic agents, angiogenic factors, regulators of cell proliferation and differentiation, and proteinase inhibitor [15, 16, 20–23]. They are able to trigger antigen-driven immune response, acting as interplayer between innate and adaptive immune systems. In the view of more functions of AMPs than the antimicrobial activity the alternative term describing these molecules has been proposed—alarmins [23]. AMPs may influence cells through several mechanisms: direct binding to specific receptors, changing the membrane domain of receptors without acting as a ligand, or stimulation of the release of membrane bound growth factors [15, 19]. Recent advances in our understanding of the novel functions of AMPs have opened new perspective on the functioning of immune system and shown the association between their altered production and various human diseases. AMPs have been demonstrated to be abundantly expressed in some chronic immune-mediated inflammatory diseases, such as psoriasis, and to contribute to their pathogenesis as important mediator of epidermal-dermal communication. Dysregulation of AMPs may contribute to the disease phenotype and influence T-cell mediated response and epidermal changes. Among AMPs there are three subclasses that have gained the special interest as a potentially important player in the pathogenesis of psoriasis: cathelicidin, S100 proteins, and defensins [15, 17, 19, 20].

### 1.1. Cathelicidin

In the last decades the relevance of abnormal production of cathelicidin for the development of psoriasis has been highlighted. Cathelicidins are one of the major families of AMPs with N-terminal prosequence and C-terminal antimicrobial domain, found in both vertebrates and invertebrates Cathelicidin is named from highly conserved N-terminus “cathelin” domain [10–12, 16, 25–27]. Application of mouse molecular genetic models with targeted deletion of cathelicidin showed that this AMP is essential for the normal functioning of the innate host defense [28]. Contrary to other species, in human single cathelicidin gene, cathelicidin antimicrobial peptide* CAMP*, located on chromosome 3 has been identified as a coding region for inactive precursor protein [29]. The expression of cathelicidin has been shown to be upregulated in psoriatic skin lesions [10]. Moreover, the processing of cathelicidin precursor protein is specific for psoriasis. Contrary to healthy skin, LL-37 peptide is the exclusively detectable form of cathelicidin found in psoriatic lesions with possible consequences for disease phenotype. LL-37 is the carboxy-terminal peptide derived from the cleavage of precursor protein by proteases. Its name comes from 37 amino acid residues starting with the pair of leucines; it has a positive charge and forms amphipathic, alpha-helical structure [30–33]. Keratinocytes and phagocytes serve as a main source of LL-37 in psoriatic lesions; however, it may be also produced by other cell types, such as T-cells, NK-cells, monocytes, mast cells [10, 11, 15, 16, 23, 24, 27]. LL-37 has capacity to kill variety of microbes, including* Staphylococcus aureus*,* Escherichia coli*, and* Candida albicans* that may reflect enhanced antimicrobial defense of disrupted skin barrier and lower frequency of infection in patients with psoriasis [10, 19, 34]. However, additionally to its antimicrobial capacity, LL-37 has been shown to modify host immune responses and significantly contribute to the pathogenesis of psoriasis [9, 15, 19, 20, 33, 35–37]. LL-37 has been proposed to drive autoimmune inflammatory process in psoriasis by variety of mechanisms including the peptide-nucleic acid binding phenomenon [9].

Despite the considerable progress in understanding the various aspects of psoriasis immunopathogenesis, the initial step driving the inflammatory process in psoriasis is still not fully elucidated [1–5, 8, 9]. The secretion of IFN-*α* by activated plasmacytoid dendritic cells (pDCs) is thought to be one of the earliest events in psoriasis pathogenesis that subsequently primes local innate and adaptive immune system [8]. Overexpressed in psoriatic skin lesions, LL-37 has shown to be crucial mediator of pDCs activation. By formation of aggregates with self-DNA, LL-37 enables pDCs to recognize self-DNA released from damage cells through pattern recognition receptors Toll like receptor (TLR) 9. Self-DNA-LL-37 complexes prime pDCs for production of large amount of IFN-*α* Table 1(a) [9]. This phenomenon may represent an important mechanism in which LL-37 initiates an autoinflammatory cascade in psoriasis. The stimulated DCs have capacity to influence the differentiation of naive T-cells into Th1/Th17 cells with production of associated cytokines that further drive development of psoriatic lesions. According to classical concept TLR-9 recognizes unmethylated DNA sequences found in microbial DNA and serves as an innate warning system against infections. In health pDCs are unable to recognize self-DNA [9, 15, 38]. LL-37 has been also reported to form complexes with self-RNA in psoriatic lesions leading to the activation of both pDCs and myeloid DCs (mDCs) and production of proinflammatory cytokines by these cells. Self-RNA-LL-37 complexes prime pDCs for production of IFN-*α* through TLR-7 and trigger production of tumor necrosis factor (TNF)-*α*, IL-6, by mDCs in TLR-8-mediated manner Table 1(a) [39]. Human slan (6-sulfo LacNAc) DCs and the novel subtype of DCs are also activated by complexes formed of LL-37 and self-RNA through TLR-7, TLR-8, and TLR-9. These cells have been shown to abundantly produce TNF-*α* and to induce Th1/Th17 differentiation. The stimulation of slanDCs by self-RNA-LL-37 complexes results in production of several proinflammatory cytokines by these cells Table 1(a) [40]. LL-37 has been also shown to transport self-DNA into monocytes that resulted in activation of these cells with production of IFN-*α*. The stimulation of monocytes is mediated by double-stranded DNA, independently of TLRs Table 1(a) [41]. Furthermore LL-37 has been demonstrated to prime keratinocytes for production of type I IFNs (IFN-*α* and IFN-*β*) and other proinflammatory cytokines, acting as alarmin and amplifying inflammatory process in the skin Table 1 [35, 36]. Activation of keratinocytes by LL-37 enhanced TLR-9 expression by these cells and subsequently responsiveness to self-DNA [35]. Recently Chen et al. [42] showed that LL-37 modulates proinflammatory response in human keratinocytes. LL-37 induces production of IL-6 and CXCL-8/IL-8 by human keratinocytes, acting synergistically with Th17/Th22 cytokines (IL-17 and IL-22) Table 1(a). Because of abundance of keratinocytes in psoriatic skin lesions this mechanism may have crucial relevance to the pathogenesis of psoriasis. Keratinocytes with the ability to modulate immune host response may constitute attractive target for therapeutic intervention in psoriasis [35]. LL-37 also inhibits apoptosis of keratinocytes by upregulation of cyclooxygenase-2 (COX-2) and inhibitor of apoptosis-2 (IAP-2) and primes neutrophils for production of reactive oxygen species and human alpha-defensins Table 1(a) [43, 44]. Self-DNA and RNA complexes with excessively produced LL-37 may constitute the trigger factor that initiate and drive T-cell mediated autoinflammatory process in psoriasis by activation of antigen presenting cells, phagocytes, keratinocytes, and inhibition of apoptosis of the latter cells. The epidermis is a rich source of self-nucleic acids that can be released by external stimuli, such as trauma, infection, or excessive keratinocytes differentiation. Therefore, the above mentioned mechanisms may also partly explain Koebner phenomenon [15, 19].

Cathelicidin may trigger inflammatory cell recruitment and cytokines release acting also through other mechanisms. LL-37 is a potent chemoattractant for mast cells, monocytes/macrophages, T-cells, and neutrophils acting through activation of formyl-peptide receptor-like 1 (FPRL-1) Table 1(a) [23, 24, 45]. Cathelicidin also promote neovascularization in psoriatic lesions, inducing the proliferation and migration of endothelial cells that is crucial process in formation of new blood vessels Table 1(a) [46]. Vitamin D3 has been identified as a potent inducer and regulator of LL-3. IL-17A, a key mediator in psoriasis pathogenesis, has been shown to enhance the induction of cathelicidin by vitamin D3 [47, 48]. Furthermore, the immunomodulatory function of LL-37 in psoriasis reflects its dual action on keratinocytes, both pro- and anti-inflammatory, depending on location. The extracellular or endosomal LL-37 enhances TLR9 expression in keratinocytes leading to increase in type I IFNs production; intracellular or cytosolic LL-37 blocks the DNA-triggered formation of absent in melanoma 2 in melanoma 2 (AIM2) inflammasomes in keratinocytes, inhibiting IL-1*β* release [35, 49]. The latter mechanism may explain the effectiveness of vitamin D analogues in the treatment of psoriasis that regulate keratinocytes proliferation and differentiation but increase expression of LL-37 [49]. The elevated serum levels of cathelicidin have been shown in patients with psoriasis. Cyclosporine therapy reduced cathelicidin serum level, whereas NB-UVB therapy increased both serum levels of vitamin D and LL-37 [50, 51]. The above mentioned data underlines the important role of cathelicidin in initiating and driving an autoinflammatory cascade in psoriasis.

### 1.2. S100 Proteins

In the last decades, S100 proteins have been increasingly emerging as a key player of innate immunity, important in the pathogenesis of various inflammatory, metabolic, and neoplastic disorders. S100 proteins are a multigenic family of small (9–13 kDa), acidic proteins that are characterized by the presence of two calcium binding EF-hands motifs. They are found exclusively in vertebrates and most of them are encoded within epidermal differentiation complex (EDC) on chromosome 1q21. More than twenty different types of S100 proteins have been identified so far. They are produced as monomers and spontaneously form dimers/multimers [52–54]. The tissue and cell-specific expression patterns of S100 proteins may suggest their functional complexity and diversification. They are involved in intracellular calcium-dependent and zinc-dependent signaling, regulation of cells metabolism, proliferation and differentiation, intercellular adhesion and invasion. Some AMPs have been shown to regulate inflammatory response acting through various mechanisms, including regulation of transcriptional factors, modulation of enzymatic activity, or cytoskeletal dynamics. In addition, S100A7 (psoriasin), S100A8 (calgranulin A), S100A9 (calgranulin B), S100A12 (calgranulin C), and S100A15 (koebnerisin) show antimicrobial activity [15, 52–55].

#### 1.2.1. S100A7 (Psoriasin)/S100A15 (Koebnerisin) Subfamily

Human psoriasin (S100A7) and koebnerisin (S100A15) were first identified as overexpressed in psoriatic plaques [13, 14]. They are encoded by genes located within the EDC on human chromosome 1q21 that was identified as one of psoriasis candidate loci (PSORS4). Additionally, koebnerisin reveals an unusual genomic organization and is transcribed into two alternate isoforms, S100A15-S (short isoform) and S100A15-L (long isoform); they share the same coding region but are differentially regulated through alternate promoters [14]. The main difference between these two proteins is the presence of a calcium-binding EF-hand motif in N-terminus of S100A15 that is not found in S100A7. Despite the highest homology among the S100 family (over 90% sequence identity) both proteins show expressional and functional diversity. S100A7 and S100A15 are overexpressed by the epidermal suprabasal compartment in psoriatic lesion, where both proteins show antimicrobial activity against* Escherichia coli* [56, 57]. S100A15 is additionally produced in the epidermis by basal keratinocytes, langerhans cells, and melanocytes and in the dermis by dendritic cells, smooth muscle cells, and the endothelium of blood vessels [55, 58, 59]. Upregulated in psoriatic lesions, psoriasin and koebnerisin potentiate immune-mediated inflammatory process in the skin. Both proteins act as chemoattractants for various leukocyte subsets, linking innate and adaptive immune mechanisms. S100A7-dependent signaling is mediated through the multiligand receptor for advanced glycated end products (RAGE), whereas S100A15 acts through a pertussis toxin sensitive Gi-protein-coupled receptor (GiPCR). Psoriasin has been shown to attract all three leukocytes subtypes: lymphocytes, monocytes, and granulocytes at similar concentrations. Koebnerisin acts as a chemotactic factor for myeloid cells: granulocytes and monocytes Table 1(b) [60]. Both proteins are induced by Th1-, Th17-, and Th22-derived cytokines, important in the pathogenesis of psoriasis that create characteristic psoriatic proinflammatory milieu [61, 62]. However, IL-17A, TNF-*α*, and IL-22 differentially regulate koebnerisin and psoriasin that indicate regulatory diversification of these proteins [62]. IL-17A, a crucial player in pathogenesis of psoriasis, is principal inducer of both proteins which acts synergistically with other propsoriatic cytokines to induce S100A7 and S100A15 [59, 62]. Furthermore, S100A15 and S100A7 act as alarmins to prime keratinocytes for enhanced production of proinflammatory cytokines that are crucial in development of psoriatic lesions, such as TNF-*α*, IL-6, and IL-8 Table 1(b) [62]. Both proteins amplify the inflammatory process in the skin. S100A7 and S100A15 are upregulated in psoriasis by similar mediators and synergize to promote inflammation. Thus, targeting S100A7-/S100A15-mediated inflammatory loop may have beneficial effect in the treatment of psoriasis, as shown for the vitamin D analoque calcipotriol and TNF-*α* inhibitors [61, 62].

Moreover, psoriasin has been demonstrated to regulate neutrophils functions. It activates neutrophils to produce proinflammatory cytokines and chemokines, such as IL-6, IL-8, and TNF-*α*, macrophage inflammatory protein, (MIP)-1*α*, MIP-1*β*, and stimulates generation of reactive oxygen species by these cells. Therefore psoriasin may contribute to neutrophils stimulation during inflammation Table 1(b) [57]. S100A7 promotes also angiogenesis by induction of endothelial cells proliferation Table 1(b) [63]. The positive associations between the levels of psoriasin and severity of psoriasis as well as body mass index have been found [64]. It should be pointed out that RAGE transduces signals mediated by other S100 proteins, such as S100A8, S100A9, S100A11, S100A12, and S100A13. S100-RAGE interactions activate multiple intracellular signaling pathways, including nuclear factor kappa-light-chain-enhancer of activated B cells (NF-*κ*B), activator protein 1 (AP-1), signal transducer, and activator of transcription 3 (STAT3) resulting in increased expressions of proinflammatory cytokines and cellular adhesion molecules. RAGE is expressed at relatively low level in homeostasis and its expression increases in situation of cells activation or stress. S100 protein interactions with RAGE have been implicated in many disorders, for example, in the pathogenesis of diabetes and atherosclerosis [55, 65].

The increased susceptibility to develop inflammatory lesions upon exposure to various environmental triggers (Koebner phenomenon) is a hallmark of psoriatic skin [66]. As disease candidate genes, both psoriasin and koebnerisin are already constitutively upregulated in inflammation-prone psoriatic epidermis. S100A7 and S100A15 share an ancestral protein in mice, mS100a7a15 [58]. Mirroring the increased expression in psoriasis, transgenic mouse model primes the skin for inflammation and mediates an increased susceptibility to develop a psoriasis-like phenotype upon exposure to environmental stimuli [61]. The resulting immune response is dependent on RAGE and characterized by immune cell infiltration and elevated concentrations of Th1 and Th17 proinflammatory cytokines, which have been linked to the pathogenesis of psoriasis. This pathogenetic psoriasis model uses a psoriasis candidate gene to link the epidermis and innate immune system in inflammation priming, highlighting the S100A7A15-RAGE axis as a potential therapeutic target.

#### 1.2.2. Calgranulins

S100A8 (calgranulin A, migration inhibitory factor-related protein 8, MRP-8) and S100A9 (calgranulin B, MRP-9) are members of S100 protein family, mainly produced by myeloid cells such as neutrophils, monocytes, and macrophages. However, their expression may be induced in some other cell types, including keratinocytes, endothelial cells, and vascular muscle cells [67–70].

S100A8/S100A9 have been demonstrated to be excessively produced by psoriatic keratinocytes. Overexpressed in psoriatic epidermis, they have been shown to act through positive feedback loop and stimulate proliferation and growth of keratinocytes [67, 70, 71]. Contrary, it has been also suggested that upregulation of S100A8 and S100A9 may inhibit keratinocyte proliferation and survival and promote their differentiation through intracellular, calcium-dependent signaling [69, 70]. One possible explanation is that S100A8/S100A9 released to extracellular space may induce keratinocyte proliferation and abnormal differentiation, whereas intracellular form of these proteins may reduce mitotic activity of keratinocytes [69]. Benoit et al. [70] suggested that effect of S100A8/S100A9 in psoriasis is mainly dependent on extracellular signaling. Moreover, S100A8/S100A9 are involved in interactions between keratinocytes and other immune cells and contribute to the pathogenesis of psoriasis by generating specific psoriatic milieu. They prime keratinocytes for enhanced production of proinflammatory and proangiogenic cytokines, such as IL-6, IL-8, and TNF-*α*, growth-related gene product (GROs) Table 1(b) [71]. Both proteins attract immune cells to the sites of inflammation and thus contribute to development of psoriatic skin lesions. Furthermore, they promote angiogenesis by inducing endothelial cells proliferation and formation of new blood vessels Table 1(b) [71]. It has been shown that S100A8/S100A9 are involved in development of autoimmunity by induction of autoreactive CD8^+^ T-cells [72]. The elevated serum level of S100A8/S100A9 has been demonstrated, likely as a result of excessive production of these proteins in skin lesions [69]. S100A12 (calgranulin C) have been also shown to be overexpressed in psoriatic plaques; however its exact role in psoriasis need further investigation [73]. Calgranulins act through RAGE signaling [69].

### 1.3. Human Defensins

Defensins are group of AMPs with six conserved cysteine residues that form three intramolecular disulfide bridges. They are divided into *α*-, *β*-, *θ*-defensins, depending on amino acid sequences of cysteine residues and disulfide bridge alignment. Six human *α*-defensins, alternatively termed human neutrophil peptides (HNPs), have been identified. They are produced by neutrophils (HNP 1-4) and intestinal Paneth cells (HNP 5,6). HNP1-3 have been extracted from psoriatic scale [11, 15, 16, 19, 24]. Four human *β*-defensins (HBD1-4) have been identified in the human skin. They are produced mainly by epithelia of skin and respiratory tract but also by peripheral blood cells. These peptides have broad spectrum of antimicrobial activity against both gram-positive and gram-negative bacteria, fungi, and viruses that may be clinically associated with low level of skin infections in patients with psoriasis [12, 16, 74]. It has been demonstrated that *β*-defensin-1 knock-out mice showed higher mortality after influenza virus infection compared to wild-type mice [75]. HBD1 is constitutively expressed in human skin. Although HBD1 has only minor antimicrobial activity in comparison with other AMPs, when reduced by thioredoxin it becomes potent AMP [76, 77]. The expression of HBD2-4 in keratinocytes is very low in homeostasis but becomes upregulated during infection, inflammation, and wound healing. HBD2 and HBD3 have been found to be upregulated in psoriatic skin lesions [12, 74]. Their expression is induced by Th1- and Th17-derived proinflammatory cytokines, including TNF-*α*, IL-1*β*, and INF-*γ*. Additionally, IL-17A and IL-22 have been shown to stimulate expression of HBD2 [12, 74, 78]. 1,25-(OH) Vitamin D3 is another inducer of defensins expression [79, 80]. It has been demonstrated that individual high HBDs genomic copy number is associated with susceptibility to develop psoriasis [81]. Serum level of HBD-2 correlates with disease activity and has been proposed as marker of psoriasis severity [82]. However, further studies involving larger population of patients as well as commercially available sensitive ELISA tests are needed to establish HBD-2 serum level as a marker of psoriasis severity in daily practice. Furthermore, HBDs have been shown to have chemotactic effect on immune cells, in GiPCRs-mediated manner. HBD1-3 act as chemotactic factor for memory T-cells and DCs. HBD2 has additionally chemotactic effect on mast cells and neutrophils, whereas HBD3-4 attract also monocytes/macrophages Table 1(c) [23]. Furthermore, HBD2-4 act as alarmins and prime keratinocytes for enhanced production of proinflammatory cytokines and chemokines, including IL-6, IL-10, monocyte chemoattractant protein-1 (MCP-1), macrophage inflammatory protein-3*α* (MIP-3*α*), regulated upon activation normal T-cell expressed and secreted (RANTES) and interferon-gamma-inducible protein-10 (IP-10) that is mediated by GiPCR and 5-phospholipase C (PLC) Table 1(c) [83]. HBD2-4 also stimulate keratinocyte proliferation and migration [76]. Furthermore, HBD3-4 induce degranulation of mast cells and production of prostaglandin D2 and pruritogenic cytokines-IL-31 as well as increase mast cell-mediated vascular permeability in skin. This mechanism may contribute to the pruritus, reported in patients with psoriasis Table 1(c) [84, 85]. HBD-2 activates immature dendritic cells through TLR-4 dependent mechanism and induces T helper type 1 response [86]. Recently Tewary et al. [87] showed that HBD-2 and HBD-3, similarly to LL-37, activate pDCs. By formation of aggregates with self-DNA, HBD-2 and HBD-3 enable pDCs to recognize self-DNA through TLR-9 that results in production of IFN-*α* by these cells Table 1(c).

## 2. Conclusions

AMPs have emerged as an important multifunctional player in pathogenesis of psoriasis. Recent advances highlight the role of cathelicidin, S100 proteins, and defensins for disease susceptibility and manifestation of psoriasis. The functional diversity and activity of AMPs are far beyond simple antimicrobial action only. These host molecules play significant role in interactions between resident keratinocytes and skin infiltrating immune cells. They act as interplayer and link innate and adaptive immune mechanisms. Overexpressed in psoriatic lesions, they act as alarmins on keratinocytes and other immune cells and as chemoattractant for leukocytes. AMPs are able to prime keratinocytes for the enhanced production of proinflammatory mediators and potentiate inflammatory process in the skin. Furthermore, LL-37, HBD-2, and HBD-3 have been demonstrated to activate antigen presenting dendritic cells initiating early phase of inflammatory cascade in psoriasis and driving autoimmune response. Thus, AMPs may constitute potential target for therapeutic interventions in psoriasis. Current therapeutic strategies in psoriasis focus on inhibition of certain proinflammatory cytokines or pathways, crucial in pathogenesis of psoriasis. The inhibition of the early steps of inflammatory cascade in psoriasis, mediated by AMPs with subsequent suppression of Th1-/Th17-cells differentiation might constitute interesting therapeutic option. However, further investigations are required to downregulate proinflammatory functions of AMPs without inducing significant immunosuppression. As previously suggested [35], the normalization of aberrant, upregulated expression of AMPs in keratinocytes might provide a therapeutic “topical” strategy without risks of significant systemic immunosuppression.

## Figures and Tables

**Table 1a:** (a)

AMP	Abnormalities in psoriasis	Source	Target cells	Receptor	Mechanism of action in psoriasis pathohenesis
Cathelicidin (LL-37)	(i) Up-regulation in psoriatic skin lesions [10, 11] *Specific processing of precursor protein; LL-37 exclusively detectable form (ii) Elevated serum level	Mainly keratinocytes and phagocytes also T-cells, NK-cells, monocytes, mast cells	Protein-nucleic acid binding phenomenon on early step of psoriasis pathogenesis		
			^ 1^pDCs	TLR-9	Self-DNA-LL-37 complexes activate and prime pDCs for production of INF-*α* by pDCs [9]
			^ 1^pDCs	TLR-7	Self-RNA-LL-37 complexes activate and prime pDCs for production of INF-*α* [39]
			^ 2^mDCs	TLR-8	Self-RNA-LL-37 complexes activate and prime mDCs for production of TNF-*α*, IL-6 and stimulate differentiation of mDCs into mature DCs [39]
			^3^slanDCs	TLR-7, TLR-8, TLR-9	Self-RNA-LL-37 complexes activate slanDCs [40]
				TLR-7, TLR-8	Self-RNA-LL-37 complexes prime slanDCs for production of pro-inflammatory cytokines: TNF-*α*, IL-6, IL-12p70, IL-12/IL-23p40 through TLR7/8 [40]
			keratinocytes	TLR-9	LL-37 enhances TLR-9 expression by keratinocytes and responsiveness to self-DNA with subsequent production of type I IFNs [35]
					LL-37 acts synergisitically with IL-17 and IL-22 to prime keratinocytes for production of CXCL-8/IL-8 and IL-6 [36, 42]
			Monocytes	TLR-independent manner (mediated by ds-DNA)	LL-37 transports self-DNA into monocytes leading to the activation of these cells and production of IFN type I [41]
			Neutrophils	NADPH oxidase activation	Prime neutrophils for production of reactive oxygen species and human alpha-defensins [44]

	—		Monocytes/macrophages, T-cells, neutrophils, mast cells	^ 5^FPRL-1	LL-37 acts as chemotactic factor [23, 24]
			Endothelial cells	^ 5^FPRL-1	LL-37 promotes neovascularization by induction of the proliferation and migration of endothelial cells and formation of tube-like structures [46]

^1^pDCs: plasmacytoid dendritic cells; ^2^mDCs: myeloid dendritic cells; ^3^slanDC: 6-sulfoLacNAc dendritic cells; ^4^TLRs: toll-like receptors; ^5^formyl-peptide receptor-like 1 (FPRL-1).

**Table 1b:** (b)

AMP	Abnormalities in psoriasis	Source	Receptor	Target cells	Mechanism of action in psoriasis pathohenesis
Psoriasin (S100A7)	Up-regulation in psoriatic skin lesions expression in epidermal suprabasal compartment [13, 59]	Mainly keratinocytes and phagocytes	^ 1^RAGE	Monocytes, neutrophils lymphocytes	Chemoattractant [60]
				Keratinocytes	Alarmin, prime keratinocytes for enhanced production of pro-inflammatory cytokines: TNF-*α*, IL-6, IL-8 [62].
				Neutrophils	Alarmin, prime neutrophils for production of pro-inflammatory cytokines and chemokines, including: IL-6, IL-8, TNF-*α*, ^2^MIP-1*α*, ^3^MIP-1*β* and reactive oxygen species [57]
				Endothelial cells	Induction of angiogenesis [63]

Koebnerisin (S100A15)	(i) Up-regulation in psoriatic skin lesions (ii) Expression in epidermal suprabasal compartment, basal keratinocytes, melanocytes, Langerhans cells, dendritic cells, smooth muscle cells and endothelium of blood vessels [14, 59]	Mainly keratinocytes but possibly also other cells	^ 4^GiPCR	Monocytes, neutrophils	Chemoattractant [60]
				Keratinocytes	Alarmin, prime keratinocytes for enhanced production of pro-inflammatory cytokines: TNF-*α*, IL-6, IL-8 [62].

Calgranulin A/calgranulin B (S100A8/S100A9)	Up-regulation in psoriatic skin lesions [70, 71]	Phagocytes, psoriatic keratinocytes, endothelial cells, vascular muscle cells	^ 1^RAGE	Keratinocytes	(i) Alarmin, prime keratinocytes for enhanced production of pro-inflammatory and pro-angiogenic cytokines, such as: IL-6, IL-8, and TNF-*α*, ^5^GROs (ii) Stimulation of proliferation [68, 71]
				Immune cells (monocytes/macrophages, lymphocytes)	Chemoattractant [71]
				Endothelial cells	Induction of angiogenesis [71]

Calgranulin C (S100A12)	Up-regulation in psoriatic skin lesions [73]		^ 1^RAGE		

^1^RAGE: multiligand receptor for advanced glycated end products; ^2^MIP-1*α*: macrophage inflammatory protein-1*α*; ^3^MIP-1*β*: macrophage inflammatory protein-1*β*; ^4^Gi-protein-coupled receptor (GiPCR); ^5^GROs: growth-related gene product.

**Table 1c:** (c)

AMP	Abnormalities in psoriasis	Source		Receptor	Target cells	Mechanism of action in psoriasis pathogenesis
Human *β*-defensins 1, 2, 3 and 4 (HBD1–4)	Up-regulation in psoriatic skin lesions	Keratinocytes and phagocytes	HBD1–3	^ 1^GiPCR	Memory T-cells, dendritic cells	Chemoattractant [23]
			HBD2	^ 1^GiPCR	Neutrophils mast cells	
			HBD3-4	^ 1^GiPCR	Monocytes/macrophages	
			HBD2–4	^ 1^GiPCR, ^2^PLC	Keratinocytes	Alarmin, prime keratinocytes for enhanced production of pro-inflammatory cytokines and chemokines, including: IL-6, IL-10, ^3^MCP-1, ^4^MIP-3*α*, ^5^RANTES, ^6^IP-10 [83]
				^ 7^EGFR-, ^8^STAT1- ^9^STAT3-mediated		Stimulate proliferation and migration [83]
			HBD2	TLR4	Immature dendritic cells	Stimulation, induction of Th1-mediated response [86]
			HBD3-4	TLR-9	pDCS	Activation and production of IFNs [87]
			HBD3-4	^ 10^MAPK p38-and ^11^ERK1/2-mediated	Mast cells	Degranulation of mast cells, production of prostaglandin D2; increase mast cell-mediated vascular permeability in skin [76] production of pruritogenic cytokines-IL-31 [85].

^1^GiPCR: Gi-protein-coupled receptor; ^2^phospholipase C (PLC); ^3^MCP-1: monocyte chemoattractant protein-1; ^4^MIP-3*α*: macrophage inflammatory protein-3*α*; ^5^RANTES: regulated upon activation normal T cell expressed and secreted; ^6^IP-10: interferon-gamma-inducible protein-10; ^7^EGFR: epidermal growth factor receptor; ^8^STAT1: signal transducer and activator of transcription 1; ^9^STAT3: signal transducer and activator of transcription 3; ^10^MAPKp38: p38 mitogen-activated protein kinases; ^11^ERKs; extracellular-signal-regulated kinases.
